# Minimally Invasive Endovascular Repair for Nondissected Ascending Aortic Disease: A Systematic Review

**DOI:** 10.1155/2023/5592622

**Published:** 2023-09-19

**Authors:** Weixue Huo, Mengwei He, Xianhao Bao, Ye Lu, Wen Tian, Jiaxuan Feng, Zhaoxiang Zeng, Rui Feng

**Affiliations:** ^1^Department of Vascular Surgery, Shanghai General Hospital, Shanghai Jiaotong University, Shanghai, China; ^2^Department of Vascular Surgery, Changhai Hospital, Navy Medical University, Shanghai, China

## Abstract

**Objective:**

The aim of this study is to evaluate the efficacy of endovascular treatment for nondissected diseases of the ascending aorta. *Data Sources*. PubMed, Embase, and SciELO. *Review Methods*. In this study, we conducted a search on the PubMed, Embase, and SciELO databases for all cases of ascending aortic endovascular repair included in the literature published between January 2007 and July 2023, excluding type A aortic dissection. We reviewed 56 case reports and 7 observational studies included in this study, assessing the techniques, equipment, procedural steps, and results. We summarized the age, complications, follow-up time, and access route.

**Results:**

This study includes 63 articles reporting 105 patients (mean age: 64.96 ± 17.08 years) who received endovascular repair for nondissected ascending aortic disease. The types of disease include aneurysm (*N* = 16), pseudoaneurysm (*N* = 71), penetrating aortic ulcer (*N* = 10), intramural hematoma (*N* = 2), thrombosis (*N* = 2), iatrogenic coarctation (*N* = 1), and rupture of the aorta (*N* = 3). The success rate of surgery is 99.05% (104/105). Complications include endoleak (10.48%, 11/105), stroke (5.71%, 6/105), postoperative infection (1.91%, 2/105), acute renal failure (0.95%, 1/105), aortic rupture (0.95%, 1/105), thrombosis (0.95%, 1/105), and splenic infarction (0.95%, 1/105). Five patients required conversion to open surgery, two patients underwent endovascular reintervention, and four of these five patients underwent surgery due to endoleak. Early mortality was 2.86% (3/105).

**Conclusion:**

While the viability and results of endovascular repair for the treatment of ascending aortic disease are acknowledged in some circumstances, further research is needed to determine the safety and effectiveness of endovascular treatment for ascending aortic disease.

## 1. Introduction

Ascending aortic disease encompasses a spectrum of conditions, such as true aneurysm, pseudoaneurysm, intramural hematoma, and penetrating ulcer. These diseases are capable of causing severe morbidity and mortality and thus necessitate timely and appropriate conservative or surgical management. In the current clinical landscape, open surgical repair remains the gold standard intervention for mitigating the progression of these conditions and preserving the wellbeing of affected individuals.

Standard surgical treatment has good outcomes and low mortality. However, the presence of intraoperative adhesions and possible severe bleeding during sternostomy may complicate the surgical procedure and increase the risk of death [1]. As a result, complications and mortality from open surgery remain high in elderly patients with severe comorbidities or those with a history of cardiovascular surgery [2].

In a recent report on the treatment of ascending aortic pseudoaneurysm, the in-hospital mortality rate corrected by surgical ranged from 6.7% to 41%, while the survival rates at 1, 5, and 10 years after surgery were 94%, 79%, and 68%, respectively [3]. Elderly patients, inefficiency of chronic kidney disease, and previous cardiac surgery are independent predictors of increased early mortality.

Endovascular repair is a possible alternative therapy for aortic disease with fewer side effects. In addition, this method has been applied in a single or many case reports to treat nondissected ascending aortic disease.

Published reports over the past 20 years have demonstrated that the use of covered stent grafts to treat ascending aortic disease is a potentially life-saving treatment option, giving the clinical situation time to settle and enabling more precise treatment planning.

Endovascular repair has been successfully used in some ascending aortic diseases such as ascending aortic pseudoaneurysm [4] and aortic penetrating ulcer [5].

For nondissected ascending aortic disease (ascending aortic aneurysm, pseudoaneurysm, penetrating aneurysm, penetrating ulceration, and rupture), we evaluated the current condition of endovascular repair in this study.

## 2. Methods

We conduct a search of PubMed using the following medical terms: “endovascular repair” limited to the “ascending aorta” location. Similarly, we search for terms such as “endovascular repair,” “endovascular stent grafting,” “covered stent repair,” “endovascular stent placement,” and “transcatheter covered stent delivery” limited to the “ascending aorta.” The search was limited to English-language literature from the first week of January 2007 to the first week of July 2023.

We screen studies using the Strengthening the Reporting of Observational Studies in Epidemiology (STROBE) tool for critical appraisal and summarize them based on the number of participants, methods, technical interventions, and outcomes. Two independent reviewers evaluate the potentially relevant citations identified for inclusion. For the identified literature, we first perform preliminary screening based on the title and abstract. If the relevance cannot be determined, we will assess the full text for eligibility. Any discrepancies between the two reviewers were resolved through consensus.

We collect information on the included endovascular repairs and summarized them in a table, highlighting demographic characteristics, past surgical history, intervention measures, and results.

### 2.1. Inclusion Criteria

As the initial search criteria, we choose “endovascular treatment of nondissected ascending aortic diseases.” Literatures in any language are considered, hybrid surgery, and open surgery are not included, and endovascular therapy is used in all the cases. The studies included are required to report patients' baseline health status, surgical history, postoperative complications, mortality data, the treatment method used, technical configurations of endovascular treatment, as well as other necessary follow-up and patient information.

### 2.2. Exclusion Criteria

In terms of treatment modality, articles or case reports that do not involve endovascular treatment are excluded. In addition, the endovascular treatment device is limited to covered stents, while other types of devices such as occluders and coil embolization devices are excluded. In terms of disease types, articles or case reports related to aortic dissection of the ascending aorta are excluded.

### 2.3. Data Extraction

After carefully reviewing the collected reviews or case reports, the data on aortic disease, age, gender, underlying diseases, number of cases, stent implantation type, access, follow-up, complications, mortality, causes of death, and reoperation were compiled. Continuous data such as the mean ± standard deviation are also included, and dichotomous variables are expressed as percentages.

## 3. Results

**Figure 1:**
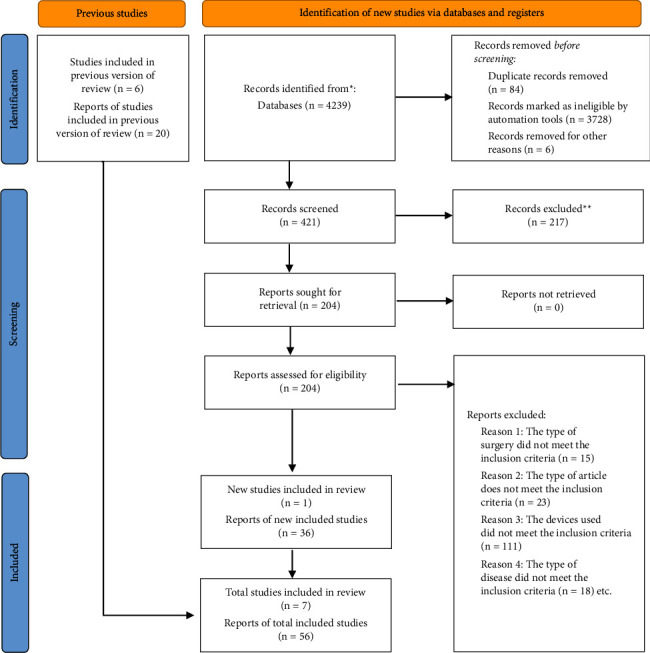
The flowchart for identifying, selecting, and eliminating acceptable studies for this review.

Figure 1 illustrates the study flowchart from the initial search of the published literature to the final inclusion or exclusion of studies. Using the search strategy outlined above, a total of 4239 articles were initially retrieved, of which 3818 were excluded based on predefined filters. Further exclusion of 217 articles is conducted based on their abstracts and titles. Among the remaining 204 articles, a full-text review was performed, resulting in a final selection of 56 case reports and 7 observational studied, for a total of 63 studies [1, 6–67]. All studies are retrospective and covered the period from January 2007 to July 2023, with a total of 105 patients described. The average age of the patients is 64.96 ± 17.08 years (range: 10–91 years), with 63 males and 41 females in the cohort. Gender is not reported in one case report, and follow-up outcome data are not reported in eight cases, accounting for a total of nine patients. The average follow-up time for the remaining 96 patients is 8.92 months. The detailed information of 105 patients in this article is shown in Table 1.

### 3.1. Indications for Ascending Aortic Endovascular Repair

**Figure 2:**
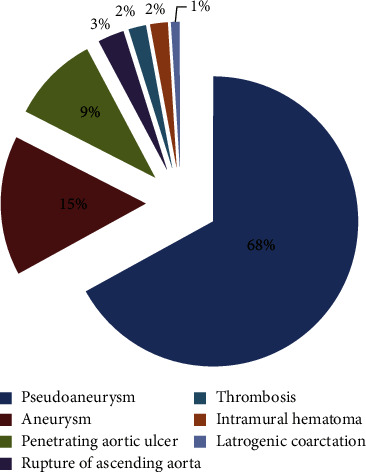
Composition of ascending aortic diseases included in this article.

Patients with aneurysms, pseudoaneurysms, penetrating aortic ulcers, intramural haematomas confined to the ascending aorta, thrombosis, iatrogenic coarctation, and aortic rupture are included in this analysis. As shown in Figure 2, the composition of each type of ascending aortic disease in this analysis is as follows: pseudoaneurysm (71 cases, 68%), aortic aneurysm (16 cases, 15%), penetrating ulcer (10 cases, 9%), aortic rupture (3 cases, 3%), thrombosis (2 cases, 2%), intramural hematoma (2 cases, 2%), and iatrogenic aortic coarctation (1 case, 1%). In all cases, aortic disease affects only the aortic segment between the sinus canal junction and the innominate artery (IA) orifice and does not involve the aortic valve and aortic root. As shown in Table 2, the vast majority of patients have a potential illness or related surgical history, so they are assessed as unsuitable for open. All patients with nondissected ascending aortic disease in this study underwent endovascular stent implantation.

### 3.2. Access

**Figure 3:**
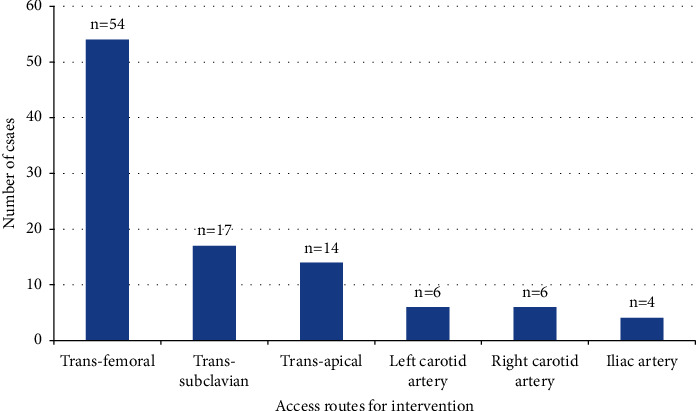
Access routes for endovascular ascending aorta repair.

In this study, the composition of the vascular approach includes the following: transfemoral artery (54 patients, 53%), subclavian artery (17 patients, 17%), transapical puncture site route (14 patients, 14%), left carotid artery (6 patients, 6%), right carotid artery (6 patients, 6%), and iliac artery (4 patients, 4%). All details are presented in Figure 3.

### 3.3. Equipment Selection and Technical Details

In addition to 3 reports that did not report the number of stents used, a total of 128 stents were used in this study, including 94 off-the-shelf stents, 20 customized stents, and 14 physician-modified stents. The average length of the stent is 74.72 ± 35.01 mm (range: 28.5–200 mm) and the average diameter is 37.16 ± 5.73 mm (range: 22−48 mm).

Of the 87 cases in which the location of the covered stent is reported, 75.9% (66 cases) of the covered stents are located proximal to the coronary artery and distal to the innominate artery. Of the composition of the distal position of the stent, 11.5% (10 cases) are located distal to the left subclavian artery, 5.7% (5 cases) covered the innominate artery, and 3.4% (3 cases) covered the left common carotid artery. And of these 17 cases, uninnominate artery stenting and left carotid stenting are also performed. It is worth mentioning that 2 patients underwent endo-Bentall surgery, which combines the repair of the aortic valve, and the proximal end of the stent is located near the coronary artery. More detailed information on the endovascular implants used is shown in Table 3.

## 4. Clinical Outcomes

As shown in Table 4, 21.9% (23/105) experience complications, including endoleak (11 cases), stroke (6 cases), postoperative infection (2 case), acute renal failure (1 case), aortic rupture (1 case), and thrombosis (1 case), splenic infarction (1 case) and 1 patient who needed to switch to open surgery. Two patients underwent endovascular reintervention and other 5 patients underwent surgery again due to endoleak.

The overall in-hospital mortality rate is 2.91% (3/105). One patient with pseudoaneurysm dies of respiratory failure and ventricular fibrillation on the 14th postoperative day. A patient with ascending aortic aneurysm develops left ventricular perforation during surgery and undergoes emergency sternotomy to fix the rupture site. But the patient still dies of multiple organ failure 24 hours later. Another patient develops an acute brainstem stroke and dies on day 15 postoperatively, and autopsy confirmed that the stent has not been displaced and the coronary orifice and brachiocephalic artery are unobstructed.

Table 5 describes the basic information of the deceased patients included in this study, including underlying diseases, surgical history, and postoperative complications. Five patients died during the follow-up period; 1 patient with aortic aneurysm died suddenly before 6 months follow-up without autopsy, one patient with a pseudoaneurysm may have died of cardiogenic stroke after stopping anticoagulation at 6 months without autopsy, one patient with aortic rupture died of multidrug-resistant pneumonia 32 days after surgery, and one patient with aortic rupture died suddenly 3 months after surgery, and autopsy determined that the cause of death is hemopericardium caused by a longitudinal tear of 4.0 cm of the ascending aortic curvature. One patient made a decision not to proceed with any further surgery and declined antibiotic therapy and died 7 months after the first endovascular stent graft procedure.

## 5. Discussion

In 2007, Lin et al. first reported a case of endovascular repair of ascending aortic nondissected disease [39]. Since then, endovascular repair has become a potential alternative for high-risk patients with nondissected ascending aortic disease [28]. In recent years, there have been increasing reports on endovascular treatment for nondissected ascending aortic diseases, but the safety and efficacy of endovascular treatment remain controversial [48]. This article summarizes a total of 105 patients who underwent endovascular repair treatment from January 2007 to July 2023. The success rate of the surgery is 99.05%, the short-term mortality rate is 2.91%, and the probability of postoperative complications is 21.90%. The results suggest that endovascular treatment for nondissected diseases of the ascending aorta is an alternative surgical approach.

## 6. Access Route

Currently, the femoral artery approach is the main route for endovascular treatment of aortic diseases. Interestingly, in this study, only 53% of the procedures are performed via the femoral artery approach, while 14% are performed via the transapical approach. Some other perspectives suggest that the transapical approach has advantages in treating nondissected ascending aortic diseases [3] despite the greater damage and need for more surgical skills. The advantage of the transapical approach is that it shortens the working distance [5], which solves the problem of inadequate length of the delivery system caused by the lack of specialized ascending aortic instruments, allowing the operator to significantly improve the manipulation of instruments and achieve more accurate landing. In addition, the transapical approach avoids the more dangerous “over-the-arch operation” and the potential risk of damaging the aortic arch branches [3, 5, 68].

### 6.1. Complications

In the treatment of nondissected ascending aortic disease, endoleak is the most common complication. The incidence of endoleak in this study report is 10.6%, including type Ia, Ib, and III. Fortunately, most endoleak are small volumes and can be treated conservatively. However, persistent endoleak, especially type I endoleak, may lead to reintervention. To maximize the proximal and distal anchoring zone and prevent endoleak, a careful preoperative examination should be done before choosing the right stent graft configuration [69]. During the postoperative follow-up stage, attention should be paid to CTA examination, timely detection, and evaluation of secondary endoleak, and treatment should be carried out [70]. In addition, stroke is also one of the common complications. In this study, its incidence rate is 5.82%. Older age, severe atherosclerosis, insufficient anticoagulation, intraoperative embolization, and left vertebral artery perfusion loss may be the important causes of stroke in patients undergoing endoaortic repair [71].

### 6.2. Devices

The design of covered stents for treating ascending aortic diseases is primarily intended for treating diseases in the descending aorta [72]. Their geometric structure is not suitable for ascending aorta. This is because the ascending aorta and aortic arch are more curved, and some patients have a conical ascending aorta, which can easily lead to endoleak and complications. Furthermore, it is worth noting that the length of the anchor zone during endovascular repair of ascending aortic disease is not clearly determined. Based on the existing descending thoracic aortic endovascular repair instruction for use (IFU), it is inferred that the proximal and distal anchorage areas of the covered stent are at least 20 mm while ensuring blood supply to the coronary artery and aortic arch branches. However, considering the short length of the ascending aorta, the anatomical differences in the distance between the sinotubular junction and the brachiocephalic artery's level of coronary artery opening, and possible accompanying aortic valve disease, this poses a significant challenge to endovascular repair [73].

In addition, the ascending aorta has a larger curvature, and the curvature on both sides of the aorta is uneven. Simple straight covered stents are only suitable for one-third of the patients, making the design of endovascular devices for the ascending aorta challenging. The recently developed Gore ascending covered stent, whose feasibility is undergoing preliminary testing in the ARISE trial, has special features that allow the covered stent to shorten its internal curvature to avoid beak-shaped bending and better adhere to the inner and outer aortic walls. As of July 2023, the results of the ARISE study are still pending (NCT02380716) [68].

So far, the vast majority of endovascular treatment cases for ascending aortic diseases have reported isolated ascending aortic lesions (not involving the aortic valve and root), located between the sinotubular junction and the origin of the brachiocephalic artery, and treated using existing endovascular implantation techniques.

In recent years, an advanced technique called endo-Bentall has been put into clinical practice. In a review published in 2018, Dr. Kreibich proposed endo-Bentall, a new technique for addressing root and proximal ascending aortic lesions [74]. In 2020, Dr. Feng and his colleagues had overcome many difficulties and verified this technique in animals. Animal experiments revealed that this technology might treat proximal ascending aorta diseases related to aortic valve damage [75].

At the same year Felipe Gaia published the first human experience with “endo-Bentall,” using valve fixation via catheterization on an ascending layered stent. In that same year, José Honório Palma and others successfully treated a 64-year-old female patient with endo-Bentall technology who had both an ascending aortic false aneurysm and aortic disease. After a 9-month follow-up, the patient was in good general condition and had no myocardial infarction. The endo-Bentall technique involves using an aortic valve stent implantation procedure to treat aortic lesions involving the valve. It has the following characteristics: an aortic valve ring, a bare area at the sinotubular junction, which is beneficial for reconstructing coronary artery blood flow, and distal anchoring at the ascending aortic level. This new technique provides new opportunities for some patients who cannot be treated by traditional methods. It should be noted that the current technique requires custom-made stents and further research is necessary, as there is no specialized device to bridge the main stent. The long-term patency and durability of the coronary artery are also questionable [76].

### 6.3. Future Outlook

Compared to the descending thoracic or descending abdominal aorta, the ascending aorta is more complex morphologically and physiologically. Therefore, compared to truncating grafts or employing longer cuffs, the design and production of endovascular repair devices for the ascending aorta is significantly more difficult. The “endo-Bentall” technique, which extends the proximal anchorage region to the aortic root and aortic annulus, presents a solution for valve performance, coronary perfusion, and treatment of ascending aortic disease. However, it should be noted that this technology is not yet fully mature, and further long-term follow-up studies and successful case outcomes are required to support its reliability.

## 7. Conclusion

In some circumstances, it has been agreed that utilizing covered stents to treat ascending aortic disease is feasible and advantageous, but more research is needed to determine whether this approach is safe and effective. In some high-risk patients with ascending aortic disease, endovascular repair will become more and more common although further research is required. In fact, there is still a need for large clinical trials and technical problems must be solved.

## Figures and Tables

**Table 1:** Case series summary of proximal endovascular aortic procedures.

Authors	Years	AA disease	Number of cases	Age (years)	Gender	Access route	Follow-up (months)	Mortality	Complications	Reintervention
Gormley et al.	2023	AAPs	1	52	F	TF	7	1 (death at 7 m)	Postoperative infection	1 endovascular redo
Kus et al.	2023	AAPs	1	67	F	TF	NR	0	No	No
Thomas et al.	2022	AAPs	1	82	F	TF	NR	0	No	No
Lauren et al.	2022	AAPs	1	78	F	RSA	1	0	1 Ia leak	No
Rohan et al.	2022	AAPs	1	70	F	LSA	6	0	No	No
Michele et al.	2022	AAPs	1	34	M	TF	0	0	Acute renal failure	No
Dimitrij et al.	2022	AAPs	1	59	M	RSA	12	0	No	No
Franklin et al.	2021	AAPs	1	62	M	TF	12	0	No	No
Roman et al.	2021	AAPs	1	73	M	TF	4	0	No	No
Sreekanth et al.	2021	PAUs	1	57	F	TF	6	1 (death at 6 m)	1 stroke	No
Antonio et al.	2020	AAPs	1	10	M	LCCA	5	0	No	No
Shintaro et al.	2020	AAPs	1	82	F	LSA	3	0	No	No
Fillippos et al.	2020	AAPs	1	62	M	NR	NR	0	No	1 surgical redo
Naveedi et al.	2020	Ascending aorta rupture	1	91	F	NR	3	0	No	No
Diego et al.	2020	Aneurysm	1	64	F	TA	NR	0	No	No
Harsh et al.	2019	AAPs	1	37	F	TF	3	0	No	No
Abdelkader et al.	2019	Aneurysm	1	79	M	TF	2	0	No	No
Abdelkader et al.	2019	Aneurysm	1	55	M	TF	3	0	No	No
Jassica et al.	2019	AAPs	1	77	F	RCCA	4	0	No	No
Alexzander et al.	2019	Aneurysm	1	85	F	Iliac	NR	0	No	No
John et al.	2019	AAPs	1	37	M	TF	8	0	No	No
Josephine et al.	2019	PAUs	1	79	M	TA	8	0	1 stroke	No
Bruno et al.	2018	PAUs	1	91	F	TF	1	0	No	No
Joshua et al.	2018	Ascending aorta rupture	1	56	M	TF	1	1 (death at 1 m)	Postoperative infection	No
Theodorus et al.	2018	AAPs	1	74	F	NR	3	1 (death at 3 m)	Aortic rupture	No
Liam et al.	2018	AAPs	1	NR	F	TF	6	0	No	No
Theodoros et al.	2017	PAUs	1	71	M	TF	1	0	No	No
Wanchi et al.	2017	AAPs	1	77	F	TF	12	0	No	No
Foeke et al.	2017	AAPs	1	74	F	NR	1	0	Thrombus	No
Kahlberg et al.	2016	AA thrombosis	1	64	M	TF	6	0	No	No
Sakata et al.	2016	AAPs	1	17	F	TF	12	0	No	No
Khoynezhad et al.	2016	AAPs/PAUs	3\1	77, 84, 67/52	M3/F1	TF4	17.5	0	1 Ia leak/1 stroke	No
Howell et al.	2016	AAPs	1	78	F	TA	6	0	1 Ia leak	No
Wada et al.	2016	AAPs	1	42	M	LCCA	6	0	1 Ib leak	No
Piffaretti et al.	2016	AAPs/PAUs	5\3	51, 71, 73, 65, 75/73, 81, 70	M6/F2	TF5/LSA2/RSA1	40	0	1 III leak	No
Khashram et al.	2016	AAPs	1	59	M	TF	5	0	No	No
Gun Kim et al.	2016	AAPs	1	74	M	TF	1	0	No	No
Ahmad et al.	2015	Aneurysm	1	26	F	TA	12	0	No	No
Vallabhajosyula et al.	2015	AAPs/AAD	3	78, 54, 16	M3	TA2/LCCA1	33	0	No	No
Shults et al.	2015	AAPs	1	62	M	TA	7	0	No	1 surgical redo
Roselli et al.	2015	AAPs	9	38, 84, 63, 55, 73, 63, 64, 88, 61	M7/F2	TF4/TA1/TAX4	12	0	1 leak	2 surgical redo, 1 endovascular redo
Allen et al.	2015	PAUs	1	88	F	TA	2	0	No	No
Engelbert et al.	2015	AAPs	1	60	M	RSA	24	0	No	No
Oderich et al.	2015	Aneurysm	1	48	M	TF	6	0	No	No
Piffaretti et al.	2015	AAPs	1	57	M	TF	24	0	No	No
Preventza et al.	2014	AAPs	7	22, 59, 84, 69, 79, 82, 64	M5/F2	TF5/TAX1/Iliac1	14.4	1 (in hospital)	1 conversion to open surgery	No
Shaikhrezai et al.	2013	Aneurysm	2	59, 77	M2	TF2	66	0	No	No
Krankenberg et al.	2013	Aneurysm	1	67	M	TA	6	1 (death at 6 m)	Stroke and splenic infarction	No
Gray et al.	2012	AAPs	2	65, 69	M2	RCCA2	1	0	No	No
Joyce et al.	2012	AAPs	3	52, 43, 68	M1/F2	RCCA1/TF2	6/4/36	0	1 III leak	No
Gelpi et al.	2012	AAPs (mycotic)	1	45	M	TAX L	9	0	No	No
Vaughan-Huxley et al.	2011	AAPs (mycotic)	1	83	F	TAX R	1	0	No	No
Saadi et al.	2011	AAPs	2	56, 74	F/M	TAX/TF	NR	0	1 Ia leak	No
Kolvenbach et al.	2011	Ascending aorta disease	11	74, 71	M5/F6	Iliac 1/RCCA 2/LCCA 2/TA 2/TF 4	12.5	1 (in hospital)	1 Ia leak/1 Ib leak/1 stroke	1 surgical debranching
Zago et al.	2011	AAPs	1	74	M	TF	5	0	No	No
Nitin et al.	2011	AAPs	1	75	W	TF	12	0	1 leak	No
Gerosa et al.	2011	AAPs	1	52	M	TA	3	0	No	No
Veroux et al.	2011	AAPs	1	76	F	TF	3	0	No	No
Uchida et al.	2010	Ascending aorta rupture	1	62	F	TF	7	0	No	No
Martin Pedrosa et al.	2010	AAPs	1	59	NA	LSA	NR	0	No	No
Szeto et al.	2010	AAPs	1	78	M	TA	6	0	No	No
Yuri et al.	2010	AAPs	1	87	M	TF	NR	0	No	No
Lin et al.	2007	AAPs	1	78	M	LCCA	1	0	No	No
Mussa et al.	2007	AAPs	1	82	F	Iliac	0	1 (in hospital)	1 stroke	No

Values are expressed as numbers and % or the mean ± SD. AAPs: ascending aorta pseudoaneurysms; PAUs: penetrating aortic ulcers; NR: no report; M: male; F: female; R (L) CCA: right (left) common carotid artery; R (L) SA: right (left) subclavian artery; TA: transapical; TF: transfemoral; TAX: transaxillary.

**Table 2:** Preoperative characteristics (*n* = 105 patients).

Characteristics	Values
Mean age, years (range)	64.96 ± 17.08
Male gender	63 (60.6%)^*∗*^
Hypertension	78 (74.3%)
Coarctation present	14 (13.6%)
Aortic valve dysfunction	15 (14.2%)
Atrial fibrillation	11 (10.7%)
Myocardial infarction	9 (8.7%)
Heart failure	8 (7.8%)
Hyperlipidemia	8 (7.8%)
Emphysema	6 (5.8%)
Chronic rheumatic heart disease	3 (2.9%)
Ventricular fibrillation	3 (2.9%)
COPD	3 (2.9%)
Pulmonary hypertension	3 (2.9%)
Endocarditis	2 (1.9%)
Diabetes	2 (1.9%)
Persistent truncus arteriosus type 1	1 (0.9%)
Previous cerebral ischemia	1 (0.9%)
*Associated congenital heart disease*	
Marfan syndrome	2 (1.9%)
Familial dilated cardiomyopathy	6 (5.8%)
Congenital aortic stenosis	3 (2.9%)
Ventricular septal defect	1 (0.9%)
*History of operation*	
AVR	36 (34.9%)
Thoracic aortic surgery	32 (31.1%)
Open heart surgery	25 (24.3%)
Mitral valve repair	13 (12.6%)
ICD	11 (10.7%)
Median sternotomy	9 (8.7%)
Coronary artery bypass	7 (6.9%)
Heart stent implantation	5 (4.9%)
Heart transplant	2 (1.9%)
Dual chamber pacing	1 (0.9%)

Values are expressed as numbers and % or the mean ± SD. COPD: chronic obstructive pulmonary disease; AVR: aortic valve replacement; ICD: implantable cardioverter-defibrillator. ^*∗*^: when calculating gender, the number of people is calculated as 104.

**Table 3:** Technical details of the devices used for proximal endovascular aortic procedures and data of ascending aorta anatomy.

Cases	Stent graft	Number of endografts	Proximal position	Distal position	Length (mm)	Size (mm)	Coronary to IA (mm)	AA diameter (mm)	STJ diameter (mm)	Distal AA diameter mm)
AAPs	OS	2	DOCA	POIA	55/93	46*∗*46/46*∗*46		43		
AAPs	MD	1	DOCA	DOLSA	150	34*∗*30				
AA thrombosis	CM	1	DOCA	POIA	56			39		
AAPs	OS	1	DOCA	POIA	28.5	33*∗*28.5	33			
PAU	CM	1	DOCA	POIA	60	38*∗*60		37.2		
AAPs	CM	1	DOCA	POIA	80	36*∗*80		54.7		
AAPs	CM	1	DOCA	POIA	60	34*∗*60		37.4		
AAPs	CM	1	DOCA	POIA	60	40*∗*60		94.1		
AAPs	OS	2	DOCA	POIA						
AAPs	MD	1				45*∗*100				
AAPs	CM	1	DOCA	POIA	65	30*∗*65	70	26		
AAPs	OS	3	DOCA	POIA	45	36*∗*45	80	30		
PAU	OS	1	DOCA	POIA	50	45*∗*50	75	35		
PAU	OS	1	DOCA	POIA	60	45*∗*60	83	41		
AAPs	OS	2	DOCA	POIA	60	45*∗*60	90	37		
AAPs	OS	1	DOCA	POIA	70	45*∗*70	80	39		
AAPs	CM	1	DOCA	POIA	70	38–48*∗*70	70	22		
PAU	OS	4	DOCA	POIA	45	36*∗*45	48	29		
AAA	CM	2	DOCA	POIA	67	36*∗*67/38*∗*67				
AAPs	CM	2	DOCA	POIA	77	38*∗*77				
AAPs	CM	2	DOCA	POIA	77	38*∗*77				
AAPs	OS	1	DOCA	COIA	55	22*∗*55				
AAPs	MD/OS	2	DOCA	POIA	70/52	40*∗*70/46*∗*52				
AAPs (9 cases)							90.3	62	36	50
PAU + AS	MD/OS	2	DOCA	POIA	80/100	46*∗*80/45*∗*100				
AAPs	OS	2	DOCA	COIA	33	28*∗*33				
AAPs	OS	1	DOCA	POIA	100	34*∗*100				
AAPs	OS	2	DOCA	POIA	75/100	36*∗*75/37*∗*100				
AAPs	OS	1	DOCA	POIA	100	28*∗*100				
AAPs	OS	2	DOCA	COIA and 2COLCCA	100	40*∗*100				
AAPs	OS	1	DOCA	POIA	100	40*∗*100				
AAPs	OS	2	DOCA	POIA	45	32*∗*45				
AAA	CM	2	DOVG	POIA	42	34*∗*42				
AAA	CM	1	DOVG	POIA	44	38*∗*44				
AAA	OS	2	DOCA	DOLSA	150	46*∗*150/46*∗*150				
AAPs	OS	1	DOCA	POIA	45	34*∗*45	78			
AAPs	OS	2	DOCA	POIA	45	32*∗*45	85	25	28	36
AAPs	OS	1	DOCA	POIA	33	28.5*∗*33				
AAPs	OS	3	DOCA	POIA	54/33	28*∗*54/28*∗*33				
AAPs	OS	1	DOCA	POIA	64	34*∗*64				
AAPs	OS	2	DOCA	POIA	33	26*∗*33/28.5*∗*33	48	24.6		
AAPs	OS	2	DOCA	POIA		26*∗*NR				
AAPs	OS	2	DOCA	POIA	45	31.5*∗*45	85	29		
AAPs	OS	1	DOCA	POIA	100	40*∗*100	105	38		
AA disease (11 cases)			DOCA		100.9			68.1		
AAPs	OS	1	DOCA	POIA	100	40*∗*100	105	38		
AA rupture	CM	1	DOCA	DOLSA						
AAPs	OS	1	DOCA	COLCCA	100	32*∗*100				
AAPs	OS	2	DOCA	POIA	77	38*∗*77	77		31	34
AAPs	CM	1	DOCA	DOLSA			85		41	41
AAPs	OS	3	DOCA	POIA	36	32*∗*36	60	30		
AAPs	MD	2	DOCA	DOLSA	100	100*∗*40*∗*2		40.5	31.5	
AAPs	OS	1	DOCA	POIA	52	52*∗*34				
AAPs	OS	2	DOCA	POIA	52	52*∗*37*∗*2	74	30		
PAUs	MD	1	DOCA	DOLSA	200	200*∗*38*∗*30				
AAPs	OS	2	DOCA	POIA		39*∗*NR/45*∗*NR		18		
AAPs	OS	1	DOCA	POIA		32*∗*NR	59			
AAPs	OS	1	DOCA	POIA						
AAPs	OS	1	DOCA	POIA						
Aneurysm	OS	1	DOCA	POIA	80	80*∗*44				
Aneurysm	OS	1	DOCA	POIA	80	80*∗*32				
AAPs	OS	2	DOCA	POIA	45	45*∗*36*∗*2				
PAUs	OS	1	DOCA	POIA	105	105*∗*44				
Ascending aorta rupture	OS	1	DOCA	POIA		46*∗*NR				
PAUs	OS	1	DOCA	POIA	65	65*∗*38				
AAPs	MD	2	DOCA	POIA	80/60	80*∗*44/60*∗*36		31	39	
AAPs	OS	2	DOCA	DOLSA	100 ∗ 2	100*∗*37/100*∗*45				
AAPs	OS	3	DOCA	DOLSA	200/160	200*∗*36*∗*2/160*∗*36				
Aneurysm	MD	2	DOCA	POIA	70	70*∗*42*∗*2				
Ascending aorta rupture	OS	1	POCA	COIA	100	100*∗*34		30	29	
AAPs	OS	2	DOCA	POIA	90/44	90*∗*37/44*∗*37				
AAPs	OS	2	DOCA	POIA	45	36*∗*45*∗*2				
AAPs	OS	2	DOCA	POIA	50	36*∗*50*∗*2	60.5			
Aneurysm	CM	1	POCA	POIA		Endo-Bentall				
AAPs	MD	2	DOCA	POIA	60	40*∗*60				
PAUs	OS	1	DOCA	DOLSA	100	100*∗*NR	80	33		
AAPs	MD	1	DOCA	POIA						
AAPs	OS	1	DOCA	POIA	50	38*∗*50				
AAPs	OS	1	DOCA	POIA	100	45*∗*100				
AAPs	OS	2	DOCA	COIA	46 ∗ 2	36*∗*46*∗*2				
AAPs	OS	2	DOCA	DOLSA	150 ∗ 2	32*∗*150/34*∗*150				
AAPs	OS		DOCA	POIA			60	26		
AAPs	OS	2	DOCA	POIA	60 ∗ 2	46*∗*60/48*∗*60				
AAPs	OS	1	DOCA	POIA	100	40*∗*100				
Aneurysm	OS	1	DOCA	POIA		36*∗*NR				
AAPs	CM	1	DOCA	POIA	65	30*∗*65				
AAPs	OS	1	DOCA	POIA	45	32*∗*45	64			

Values are expressed as numbers and % or the mean ± SD. AAPs: ascending aorta pseudoaneurysms; PAUs: penetrating aortic ulcers; NR: no report; MD: modified design in the operating room; OS: off-the-shelf; CM: custom-made; COLCCA: covering the origin of the LCCA; LCCA: left common carotidartery; DOLSA: distally to the origin of the LSA or the proximal descending aorta; COIA: covering the origin of the IA; POIA: proximal to the origin of the innominate artery; STJ: sinotubular junction; AA: ascending aorta.

**Table 4:** The outcomes of 105 patients.

Complications	Values	
	Early outcome	During follow-up
Endoleak	7 (6.7%)	4 (3.9%)
Stroke	4 (3.9%)	2 (1.9%)
Thrombus	None	1 (0.9%)
Acute renal insufficiency	1 (0.9%)	None
Aortic rupture	None	1 (0.9%)
Conversion to open surgery	1 (0.9%)	None
Postoperative infection	2 (1.8%)	1 (0.9%)
Splenic infarction	1 (0.9%)	None
Surgical redo	5 (4.8%)	
Endovascular redo	2 (1.8%)	
Mean follow-up time	8.92 m	
30-day mortality	3 (2.9%)	

Values are expressed as numbers and % or the mean ± SD.

**Table 5:** Endovascular treatment of ascending aortic disease: death cases.

Years	Gender	Age	AA disease	Basic disease	Surgical history	Complication	Postoperative medication	Postoperative survival time and cause of death	Autopsy
2023	F	52	AAPs	Ascending aorta and a bicuspid aortic valve with moderate mixed aortic valve disease	Bentall's procedure using a 27-mm biointegral valve aortic bioconduit and mitral valve annuloplasty	Wound bleeding, hemoptysis, dizziness and empyema	Red blood cell transfusions and iron infusions/long-term antibiotics	7 m/the patient subsequently made the decision not to proceed with any further surgery and declined antibiotic therapy and died 7 months after the first endovascular stent graft procedure	No autopsy was conducted

2021	F	57	PAUs	Chronic rheumatic heart disease/ascending aortic pseudoaneurysm fistula	Mitral valve replacement surgery, open surgery using patches due to ascending aortic pseudoaneurysm	None	Aspirin Warfarin	6 m/the patient remained asymptomatic after discharge until 6 months when she died of a possible cardiogenic stroke after discontinuing anticoagulants	No autopsy was conducted

2018	M	56	AA rupture	Marfan syndrome/acute respiratory failure	Surgical history of open total arch replacement and “whole block” island reimplantation of supraaortic vessels, descending TEVAR, two remedian sternotomy to repair false aneurysms, and chest anterior transposition flap	Multiple drug resistance pneumonia	NR	32 d/died of multiple drug resistance pneumonia 32 days after operation	No autopsy was conducted

2018	F	74	AAPs	None	None	None	Metoprolol Furosemide Lisinopril	3 m/4.0 cm long longitudinal tear at the minor curvature of the aorta leading to pericardial hemorrhage	The autopsy confirmed that the cause of death was pericardial hematocele, which was a longitudinal tear 4.0 centimeters long at the small curvature of the ascending aorta without any associated dissection

2014	M	NR	AAPs	None	NR	NR	NR	14 d/sudden respiratory distress and ventricular fibrillation	No autopsy was conducted

2013	M	67	AAPs	None	Ascending aorta replacement, thoracic descending aorta covered stent implantation	Left hemisphere stroke, splenic infarction	Clopidogrel Aspirin	6 m/sudden death before follow-up CT; no autopsy was conducted	No autopsy was conducted

2011	M	74	AAPs	Myocardial infarct/severe COPD/severe addiction to smoking	None	Rupture of left ventricular wall	NR	1 d/multiple organ failure within 24 hours	No autopsy was conducted

2007	F	82	AAPs	Chronic congestive heart failure	Emergency repair of acute ascending aortic dissection	Multiple organ failure	NR	15 d/on the 15th day after surgery, the patient developed an acute brainstem stroke and died	Autopsy confirmed that the covered stent was not displaced, and the coronary artery orifice and brachiocephalic artery were unobstructed

Values are expressed as numbers and % or the mean ± SD. AAPs: ascending aorta pseudoaneurysms; PAUs: penetrating aortic ulcers; NR: no report; AA: ascending aorta.

## Data Availability

The data in this paper are all from published review or case reports, and the specific information of the paper is given in the references and Table 1.
